# Is there a link between COVID-19 and cutaneous hyperesthesia? Confirmation of a recent observation

**DOI:** 10.3205/dgkh000372

**Published:** 2021-01-12

**Authors:** Igor Alexander Harsch, Irina Atudorei, Kathrin Frank

**Affiliations:** 1 Department of Internal Medicine II, Thuringia Clinic Saalfeld “Georgius Agricola”, Saalfeld/Saale, Germany; 2 Department of Dermatology, Thuringia Clinic “Georgius Agricola”, Saalfeld/Saale, Germany

## Dear Editor,

One of the first neurological symptoms of a COVID-19 infection that has been frequently described is the loss of smell and taste. A large number of neurological problems following the infection have been reported to date [1]. As the duration of the pandemic and the number of infections increases, reports of rarer complications, possibly in connection with the disease, are also being published, for which an etiopathological connection to COVID-19 infection has not been proven, but for which the close temporal relationship nevertheless suggests causality. Cutaneous hyperesthesia was only recently reported in two patients. Krajewski et al. [2] reported on two 40-year-old patients in whom this phenomenon had occurred in immediate temporal relationship to a COVID-19 infection and lasted for about 10 days. One patient was, however, also treated with diclofenac and azithromycin. 

We describe the case of a 69-year-old female patient with a moderate [3] COVID-19 infection according to the NIH definition, who was admitted to our clinic in November 2020 and in whom the phenomenon of considerable cutaneous hypersensitivity occurred on the day of admission, especially in the abdomen and legs. Any form of touch intensified this sensation. Further neurological examination showed no pathology. Cutaneous inspection revealed no signs of shingles or other abnormalities. The patient suffered from hypertension and dyslipidemia, prior and ongoing treatment (for several years) consisted of Amlodipin, Bisoprolol, Ramipril and Simvastatin. To rule out other possible reasons for hyperesthesia, we measured the Hba1c (5.7 %) and the Vitamin B 12 level (714 ng/l, normal range 197–866 ng/l), both of which were within the normal range. The hypersensitivity spontaneously resolved 8 days later. The timeline of this symptom in our case is thus comparable to the timespan reported by Krajewski et al. [2].

This symptom seems to be rare; at the time of submission of the manuscript, 105 patients with COVID-19 infection had been treated in our clinic; no patient had previously reported this symptom. However, in more severe courses of the disease, this pathology may have been disregarded. The two cases previously reported [2] also seem to meet the NIH criteria for a “moderate” COVID-19 infection. It remains to be observed whether further case reports will be published in this regard, which would allow cutaneous hypersensitivity to be assigned to COVID-19 infection with greater certainty.

## Notes

### Competing interests

The authors declare that they have no competing interests.

### Funding

There was no financial support.

### Patient’s consent

The patient gave informed consent to the reporting of her case.
